# Supplementary material for: Band structure engineered layered metals for low-loss plasmonics
Source: Nat Commun. 2017 Apr 24;8:15133. doi: 10.1038/ncomms15133 (PMC5413982; doi:10.1038/ncomms15133)
Supplement: Supplementary Information — Supplementary Figures, Supplementary Tables and Supplementary References [file ncomms15133-s1.pdf]

Formula	Spacegroup	ICSD (ID)
$TaSe_2$	194	651950
TaS_2	194	651092
TaS_2	164	651089
$NbTe_2$	164	645529
NbS_2	160	645309
$NbSe_2$	194	645369
$SiTe_2$	164	652385
$PtTe_2$	164	649747
$PtSe_2$	164	649589
$PdTe_2$	164	649016
$NiTe_2$	164	159382
$IrTe_2$	164	33934
$RhTe_2$	164	650448
$CoTe_2$	164	625401
$HfTe_2$	164	638959
$ZrTe_2$	164	653213
$TiTe_2$	164	653071
$TiSe_2$	164	173923
$AlCl_2$	164	155670

SUPPLEMENTARY TABLE 1. **List of 19 metals from Ref.** [1] We have only included the materials which crystallize in the 1T, 2H (AB and AA') or 3R structure with spacegroups 164, 194 and 160, respectively. Note that 2H-TaS_2 stacks in the AA' structure. Note also that some materials are semiconducting in their monolayer structure but metallic in their bulk structure which would not be indicated in the data-mining paper. This transition is due to the broadening of the valence and conduction bands.

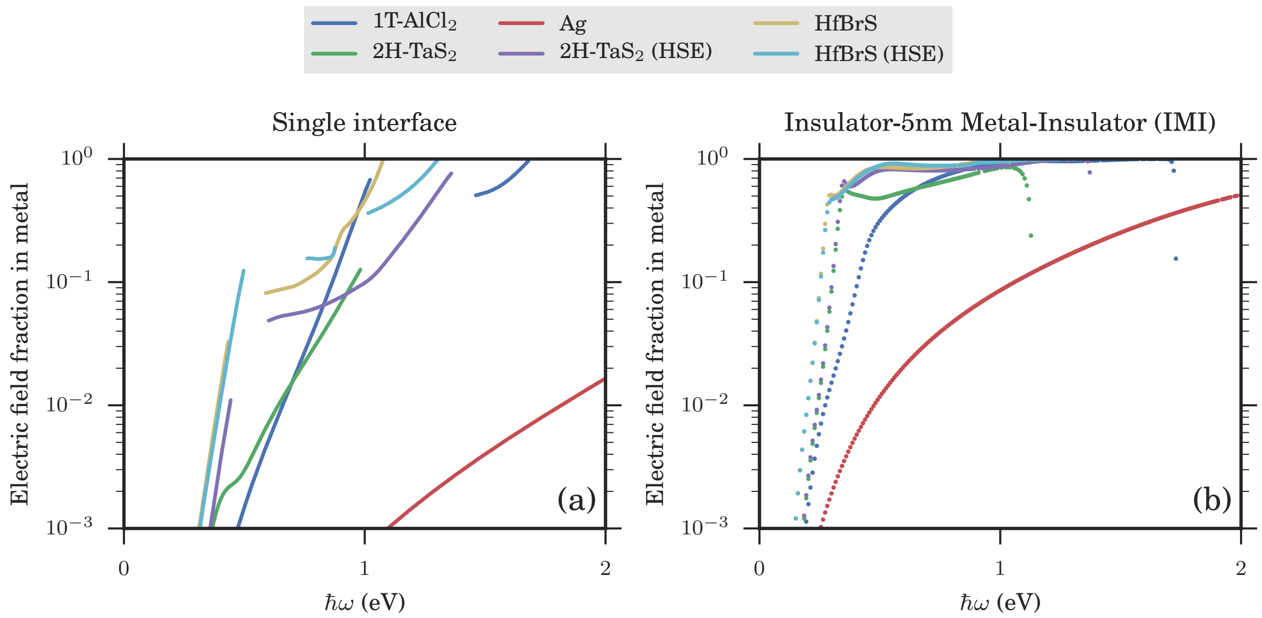

SUPPLEMENTARY FIGURE 1. **Electric field fraction in metal and insulator-metal-insulator (IMI) SPP waveguides** The fraction of the SPP electric field within the metallic structure of the single-interface SPP-waveguide and the thin-film waveguide (IMI). (a) Fraction of the electric field of a single interface SPP compared to (b) the electric field fraction of a thin film plasmon.

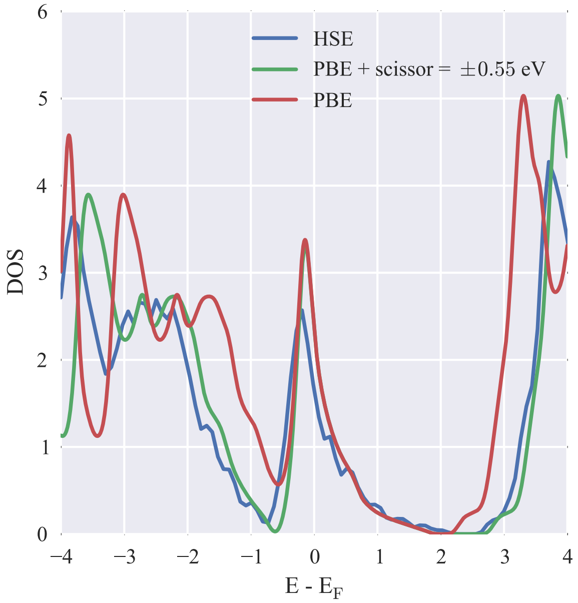

SUPPLEMENTARY FIGURE 2. **Calculated DOS for 2H-TaS$_2$ with HSE and PBE** By calculating the density of states with and without HSE06 we see that the primary effect is to increase the size of the gaps between the conduction bands and other bands. It is also seen that it is possible to reproduce the HSE06 DOS by applying a scissor operator on top of the PBE ground state. The effect of HSE on 2H-TaS$_2$ is essentially to increase the size of the gaps isolating the conduction band of 2H-TaS$_2$. We have modelled this by a scissor operator on top of PBE which is seen to reproduce the HSE-DOS well.

Formula	Hull (eV)	HOF (eV)
HfClO	-27.84	-28.58
HfClS	-19.78	-19.66
HfClSe	-18.14	-17.62
TiClO	-24.50	-28.05
TiClSe	-16.09	-16.58
ZrClO	-28.13	-30.32
TiClS	-17.43	-18.51
ZrClSe	-18.79	-19.31
ZrClS	-20.29	-21.29
HfBrO	-23.43	-25.64
HfBrSe	-13.72	-14.78
HfBrS	-15.36	-16.94
TiBrO	-21.84	-24.93
ZrBrO	-26.50	-27.45
TiBrS	-14.77	-15.69
TiBrSe	-13.44	-13.78
ZrBrSe	-17.16	-16.55
ZrBrS	-18.66	-18.55
HfIO	-22.30	-22.35
HfISe	-12.60	-12.01
TiIO	-19.63	-21.40
HfIS	-14.24	-14.05
ZrIO	-23.08	-24.29
TiIS	-12.57	-12.90
ZrIS	-15.24	-15.87
TiISe	-11.23	-10.99
ZrISe	-13.74	-13.89

SUPPLEMENTARY TABLE 2. **Stability analysis: Calculated heat of formation and hull energies for chalcogen-halogen mixtures** The stability analysis of the chalcogen-halogen mixtures as well as for the halides have been based on the material's monolayer stabilities. In this way we are restricting ourselves to materials that are stable in both monolayer and bulk phases. The heat of formation were calculated using mBEEF functional with the corrected reference energies[2]. The monolayer stabilities of the chalcogen-halogen mixtures were evaluated based on the convex hull of the competing phases. The competing phases were determined using the Open Quantum Materials Database (OQMD). The Brillouin zone of the competing bulk phases was sampled with $33a_x^{-1} \times 33a_y^{-1} \times 33a_z^{-1}$ k-point sampling where a_x, a_y and a_z represent the lattice constants in units of Angstrom. The wavefunctions were expanded on the a real space grid with the grid spacing of 0.16 Å. The halides are all stable with respect to standard phases but they are not expected to be stable with respect to the convex hull, even though we have not investigated this in detail.

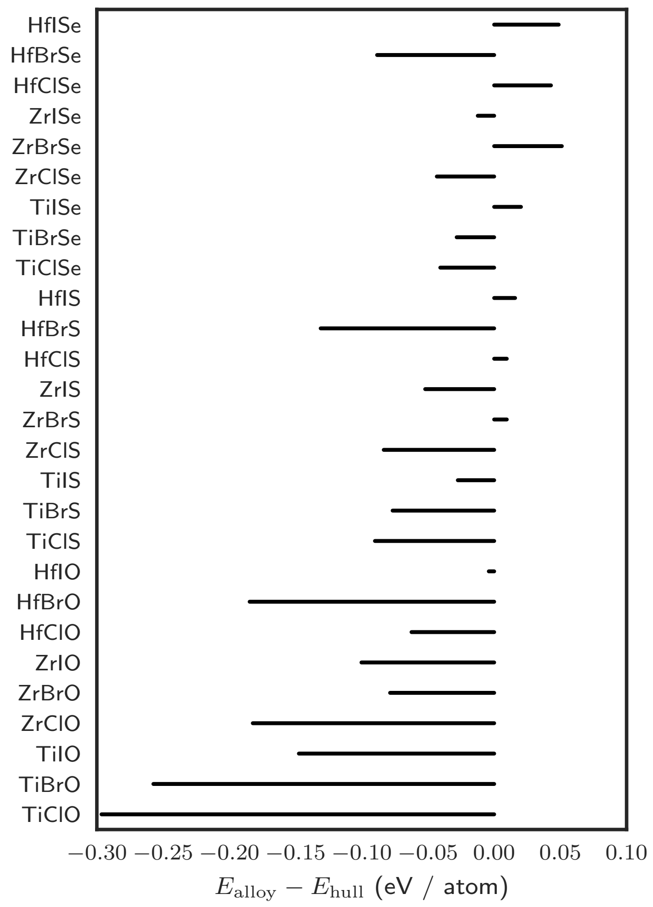

SUPPLEMENTARY FIGURE 3. **Stability of chalcogen-halogen mixtures with respect to the convex hull.** The figure is based on the data in Supplementary Figure 2.

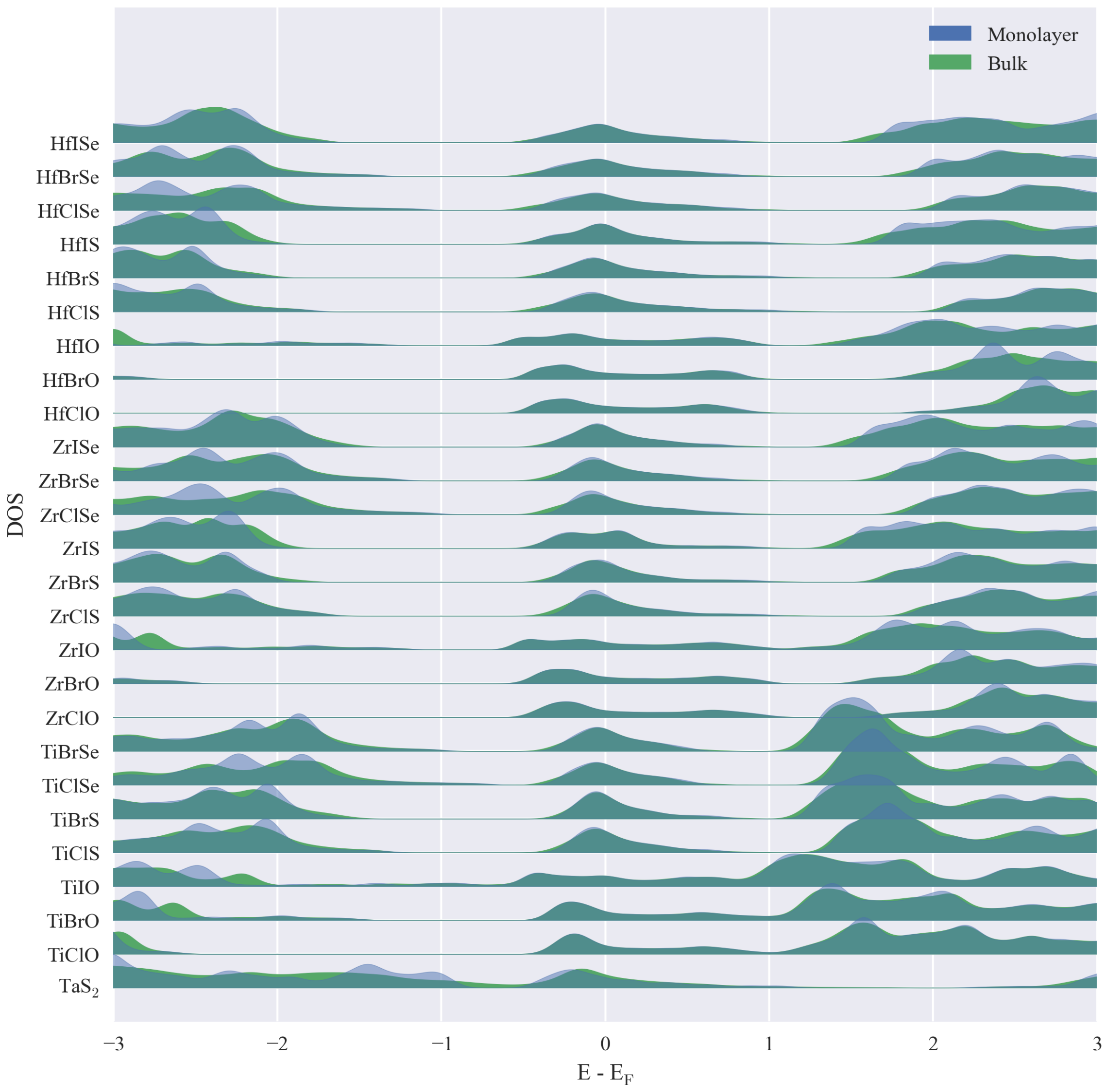

SUPPLEMENTARY FIGURE 4. **Calculated density of states for the MXY compounds** Monolayer (blue) and bulk (green) density of states for the MXY compounds. The bulk materials were relaxed with the optB88-vdW xc-functional.

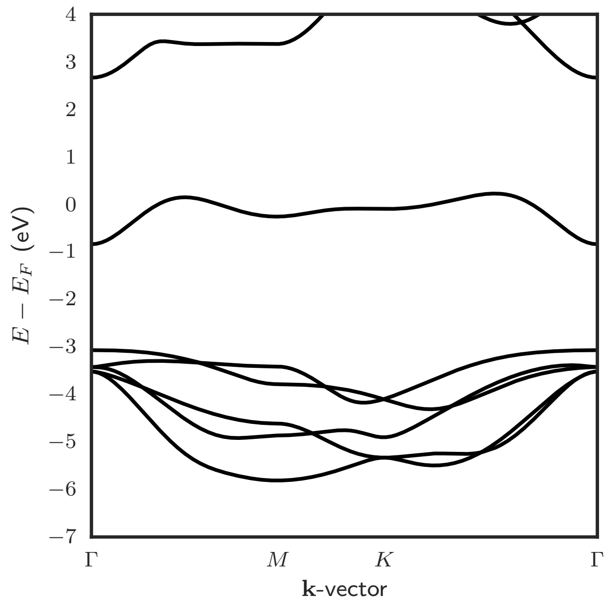

(a)

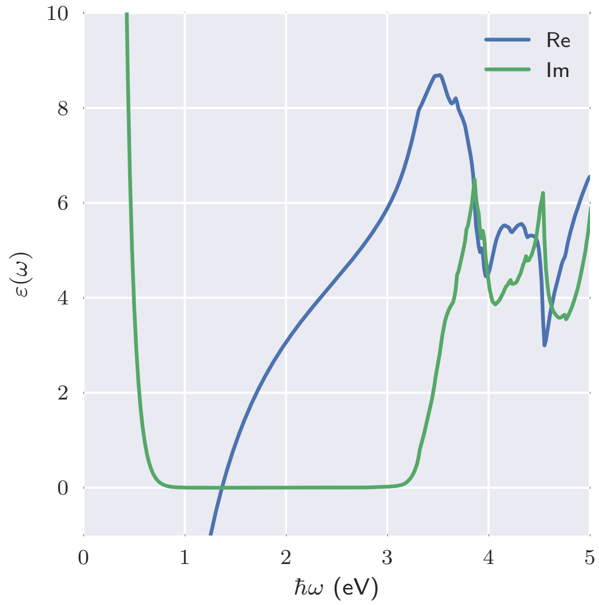

(b)

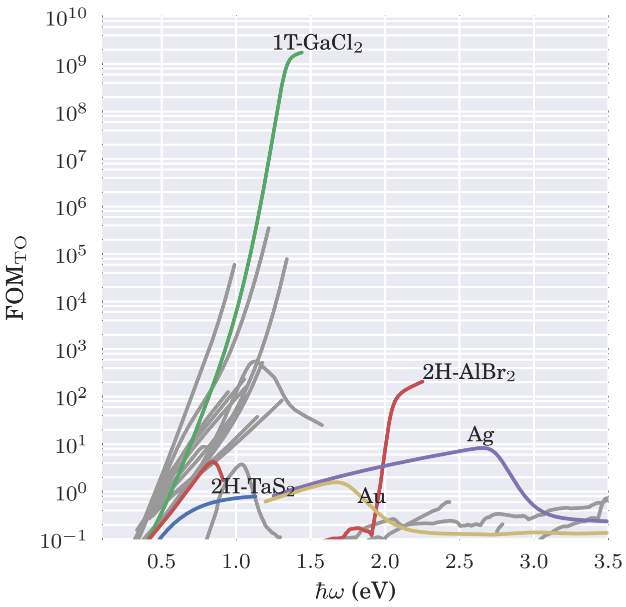

(c)

SUPPLEMENTARY FIGURE 5. **Plasmonic properties of halides** Some halides have essentially ideal bandstructures for loss-less plasmonics. For example, $GaCl_2$ has the properties necessary for a loss-less plasmonic metal. It has an intermediate band separated by large gaps which leads to a high interband onset and a sufficiently narrow conduction band (a) which limit intraband losses to lower frequencies. (b) The calculated dielectric shows that losses are essentially zero at frequencies larger than 1 eV. This is due to its special bandstructure. (c) Figure of merit for the halides for applications within transformation optics.

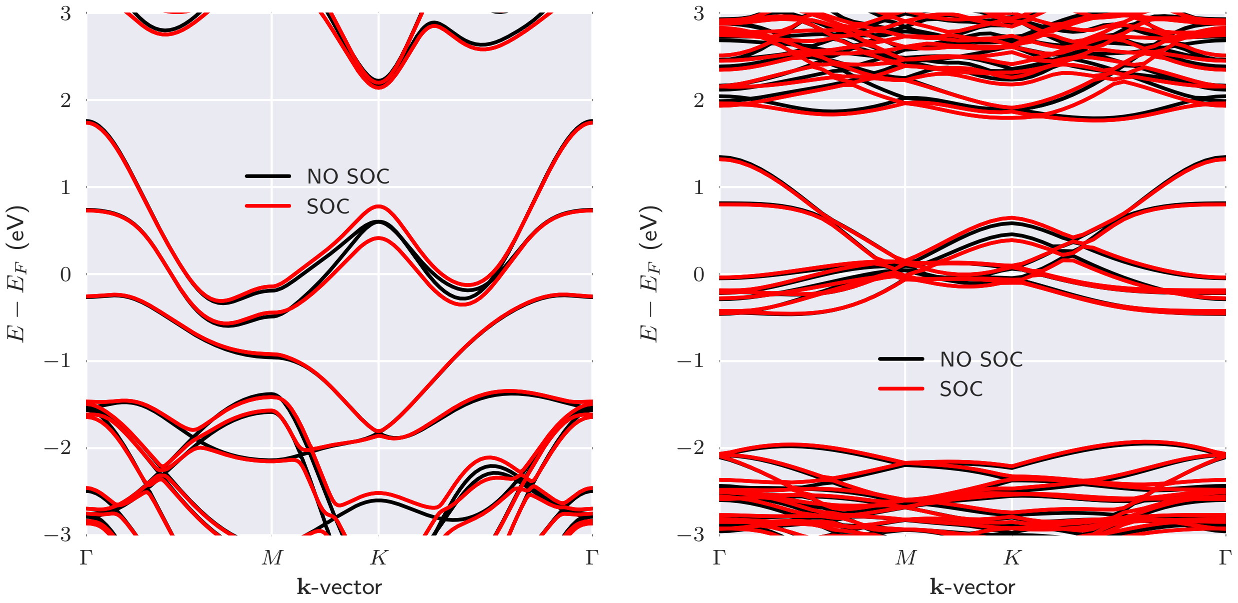

(a) (b)

SUPPLEMENTARY FIGURE 6. **Effect of spin-orbit coupling** Bandstructure of (a) 2H-TaS_2 and (b) HfBrS with and without spin-orbit coupling (SOC). The SOC was calculated non-self-consistently with GPAW which generally is found to be sufficient. The general effect is a splitting of the conduction bands at the K-point most significant for 2H-TaS_2. The bandwidth of the conduction bands is unchanged and the Fermi-velocities are essentially unchanged. From these results we conclude the SOC to be non-essential for the plasmonic properties of metals included in this study.

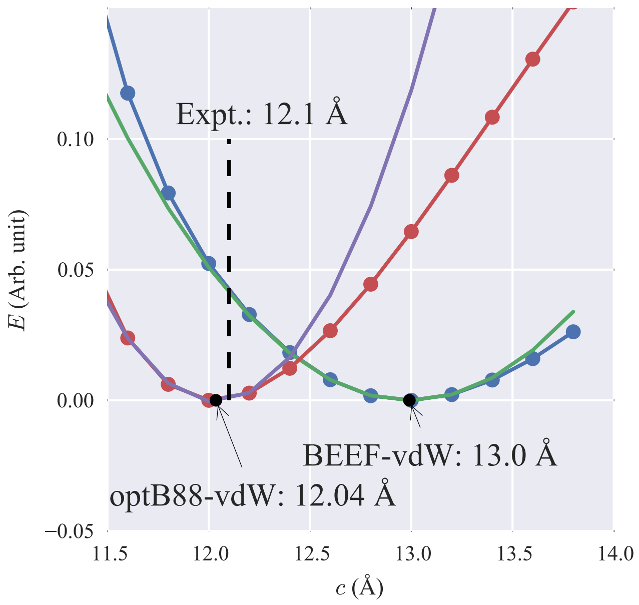

SUPPLEMENTARY FIGURE 7. **Relaxation of 2H-TaS_2 compared to experiment** Comparing the performance of the van der Waals DFT functionals optB88-vdW and BEEF-vdW for predicting the out-of-plane lattice constant of 2H-TaS_2. The relaxation was done by fixing the relative atomic positions within a layer and varying the interlayer distance. The BEEF-vdW functional overestimates the out-of-plane lattice constant by approximately 1 Åcompared to experiment, whereas the optB88-vdW functional give a more accurate description of the interlayer bonding distance.

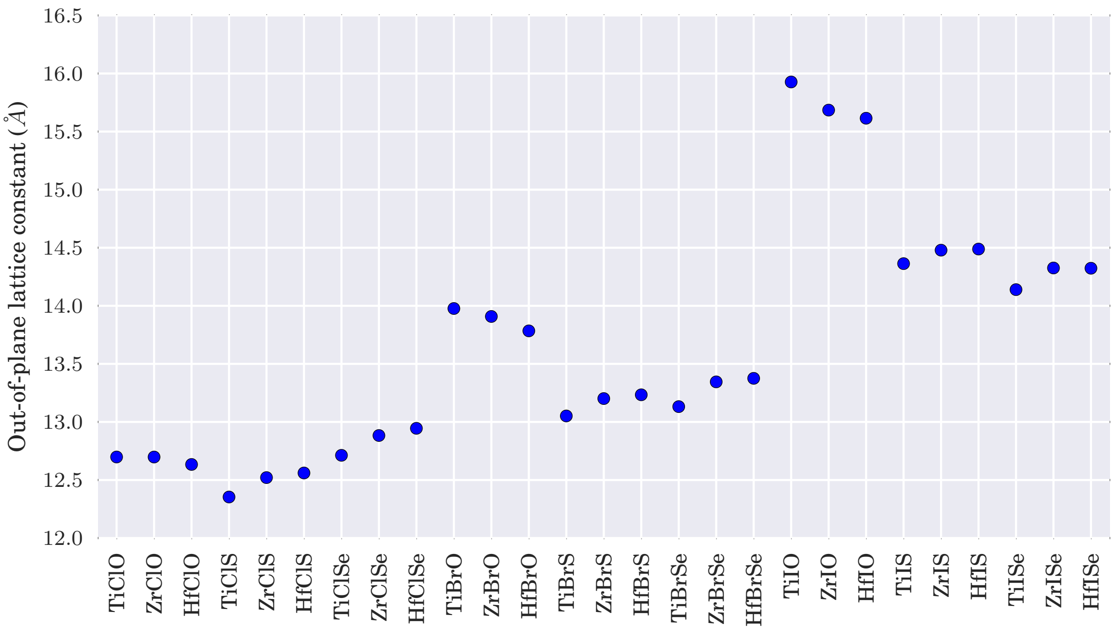

SUPPLEMENTARY FIGURE 8. **Calculated out-of-plane lattice constant for the chalcogen-halogen 2H-MXY compounds** The structures were relaxed using the optB88-vdW functional using the same procedure as described in Supplementary Figure 6.

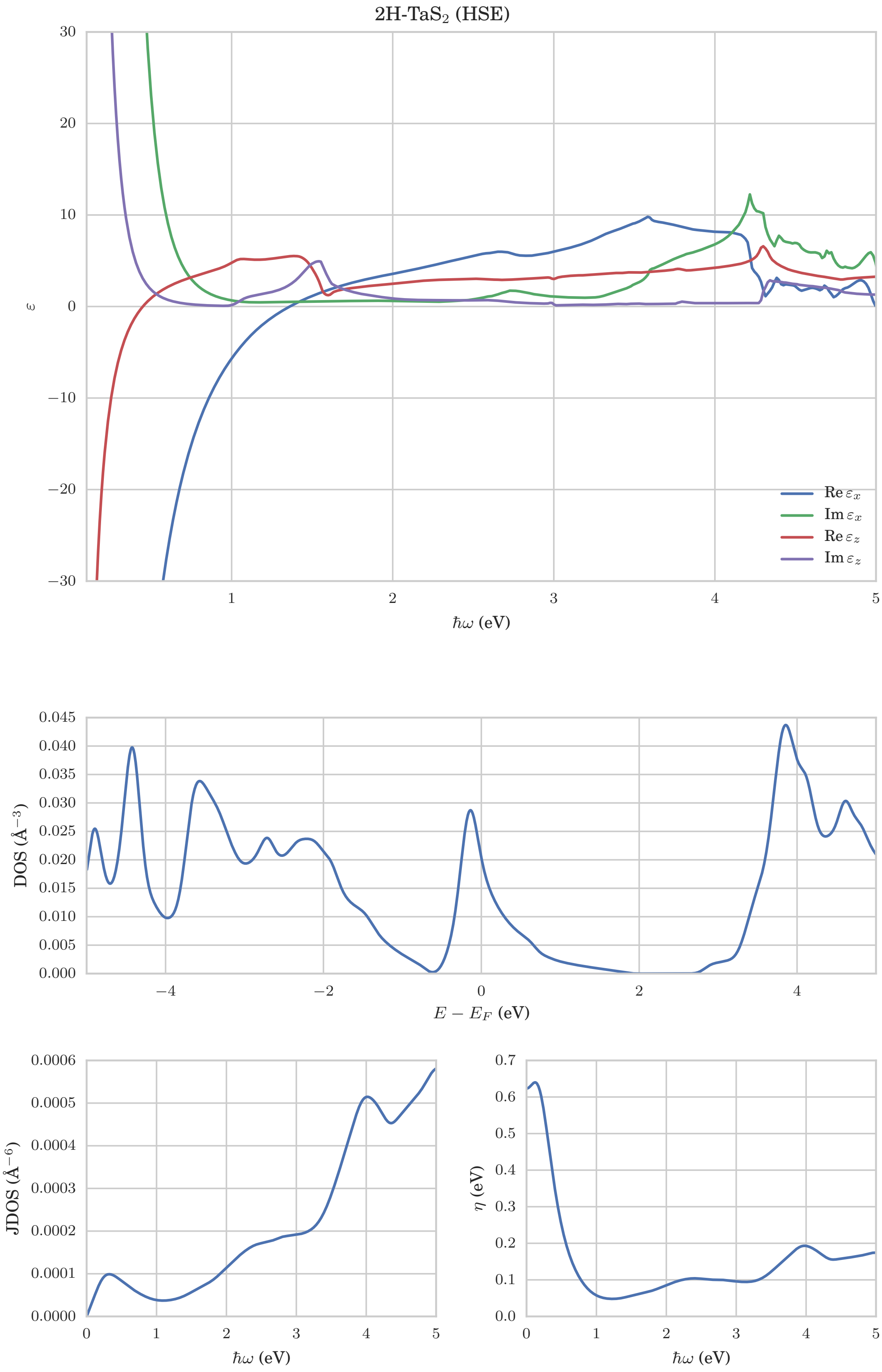

SUPPLEMENTARY FIGURE 9. **Data sheet for 2H-TaS$_2$ with HSE06 scissor operator applied** Dielectric function (ε), density of states (DOS), joint density of states (JDOS) and scattering rate (η).

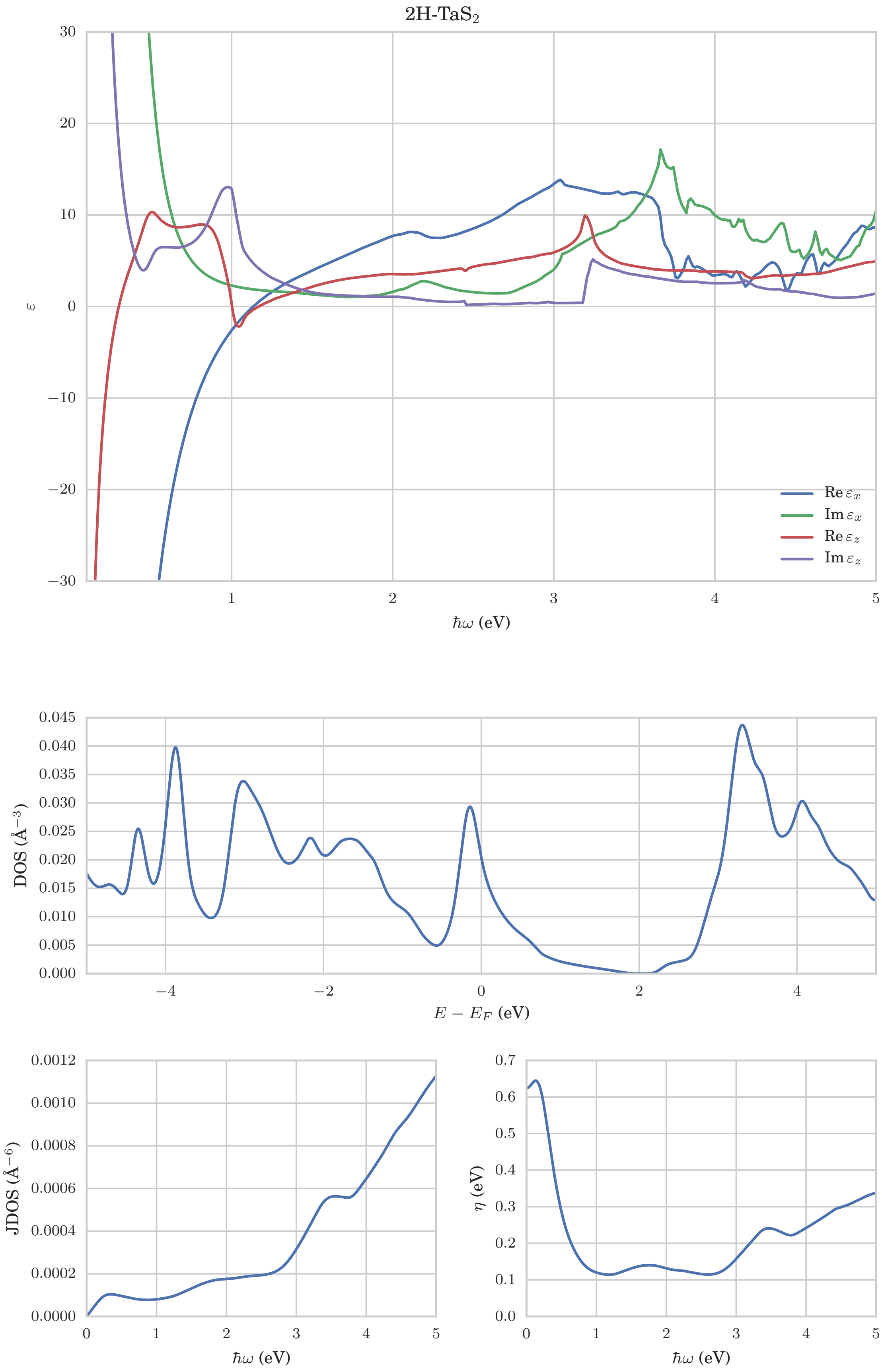

SUPPLEMENTARY FIGURE 10. **Data sheet for 2H-TaS$_2$** Dielectric function (ε), density of states (DOS), joint density of states (JDOS) and scattering rate (η).

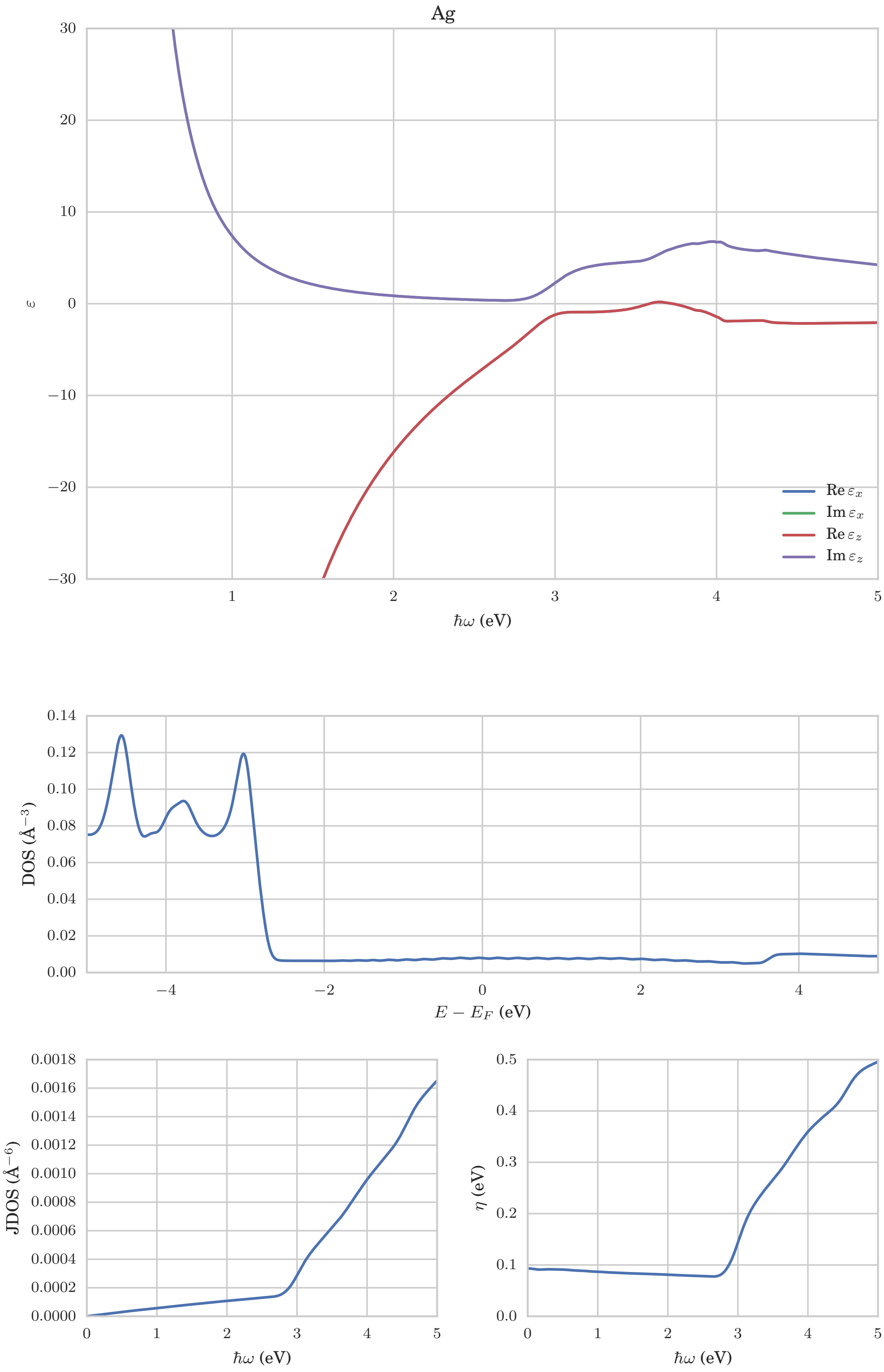

SUPPLEMENTARY FIGURE 11. **Data sheet for silver** Dielectric function (ε), density of states (DOS), joint density of states (JDOS) and scattering rate (η).

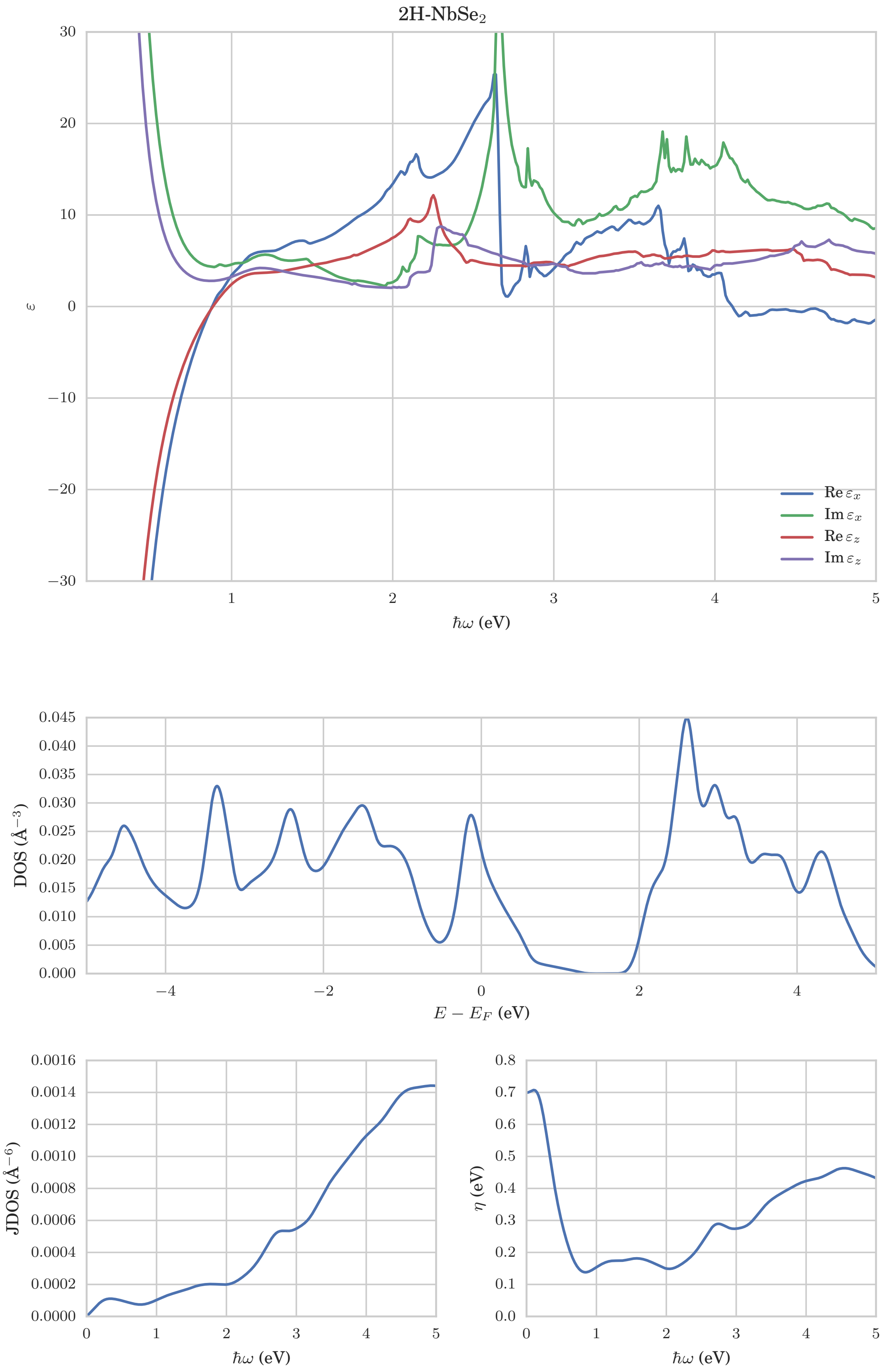

SUPPLEMENTARY FIGURE 12. **Data sheet for 2H-NbSe$_2$** Dielectric function (ε), density of states (DOS), joint density of states (JDOS) and scattering rate (η).

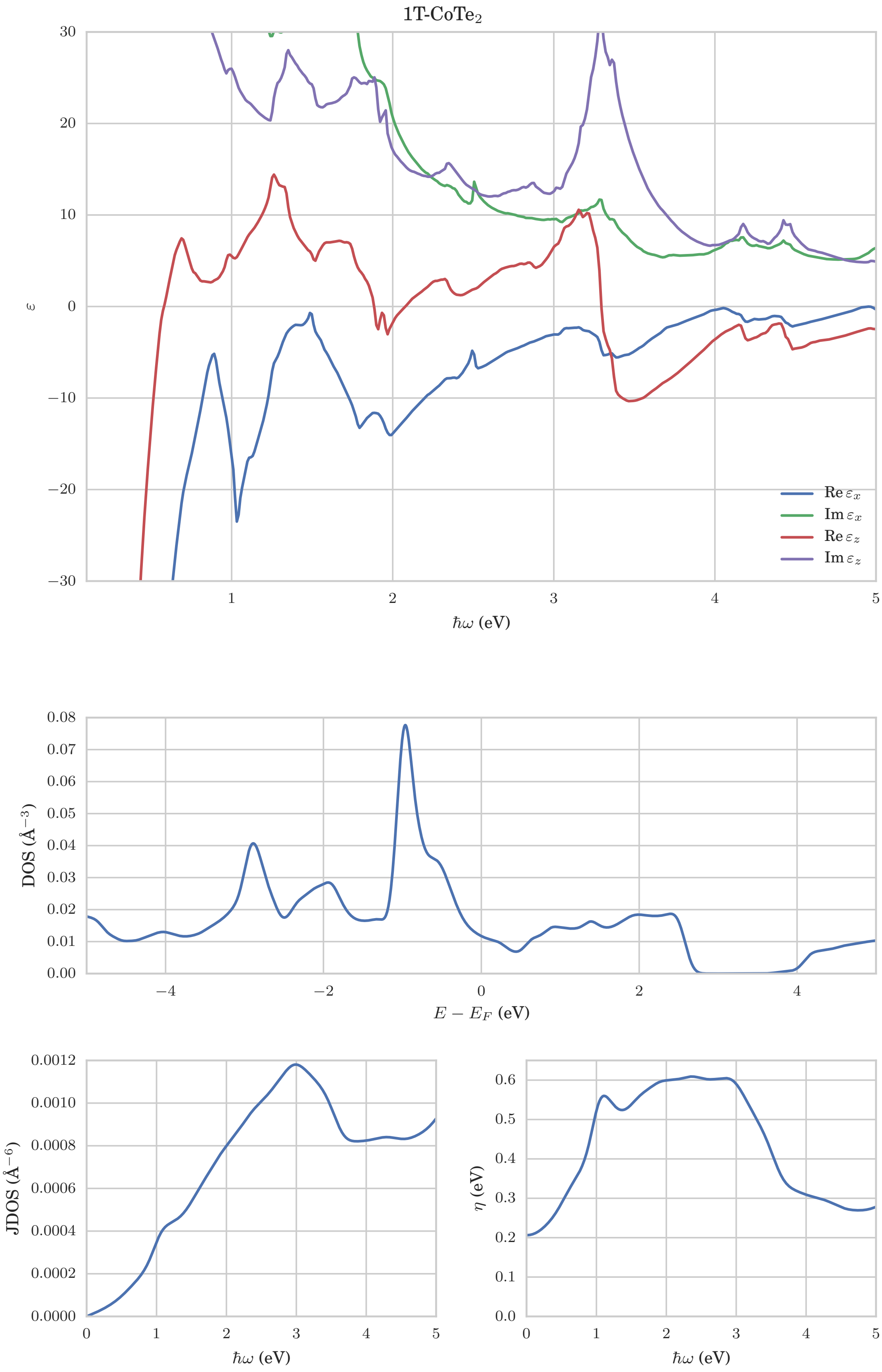

SUPPLEMENTARY FIGURE 13. **Data sheet for 1T-CoTe$_2$** Dielectric function (ε), density of states (DOS), joint density of states (JDOS) and scattering rate (η).

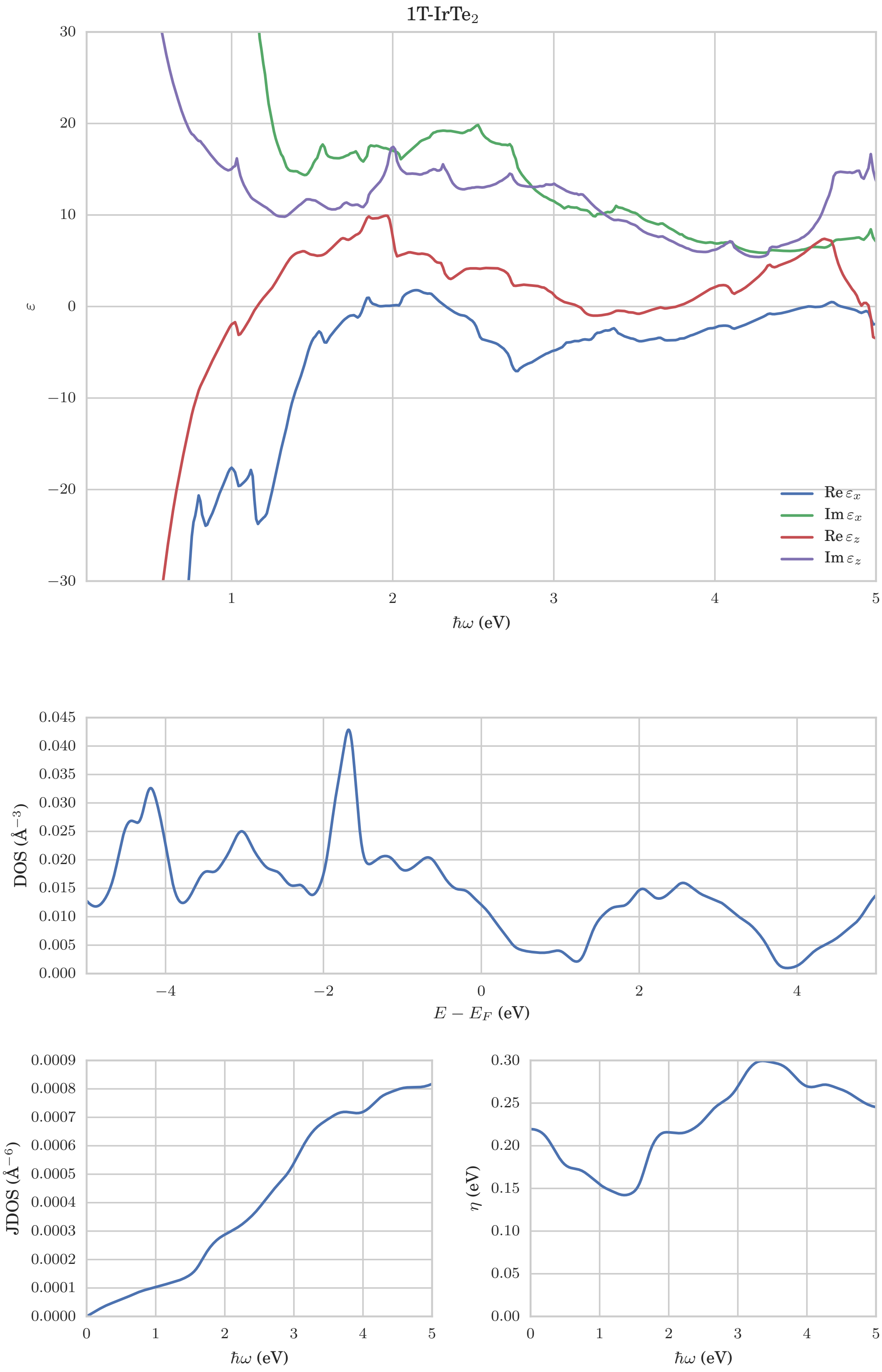

SUPPLEMENTARY FIGURE 14. **Data sheet for 1T-IrTe$_2$** Dielectric function (ε), density of states (DOS), joint density of states (JDOS) and scattering rate (η).

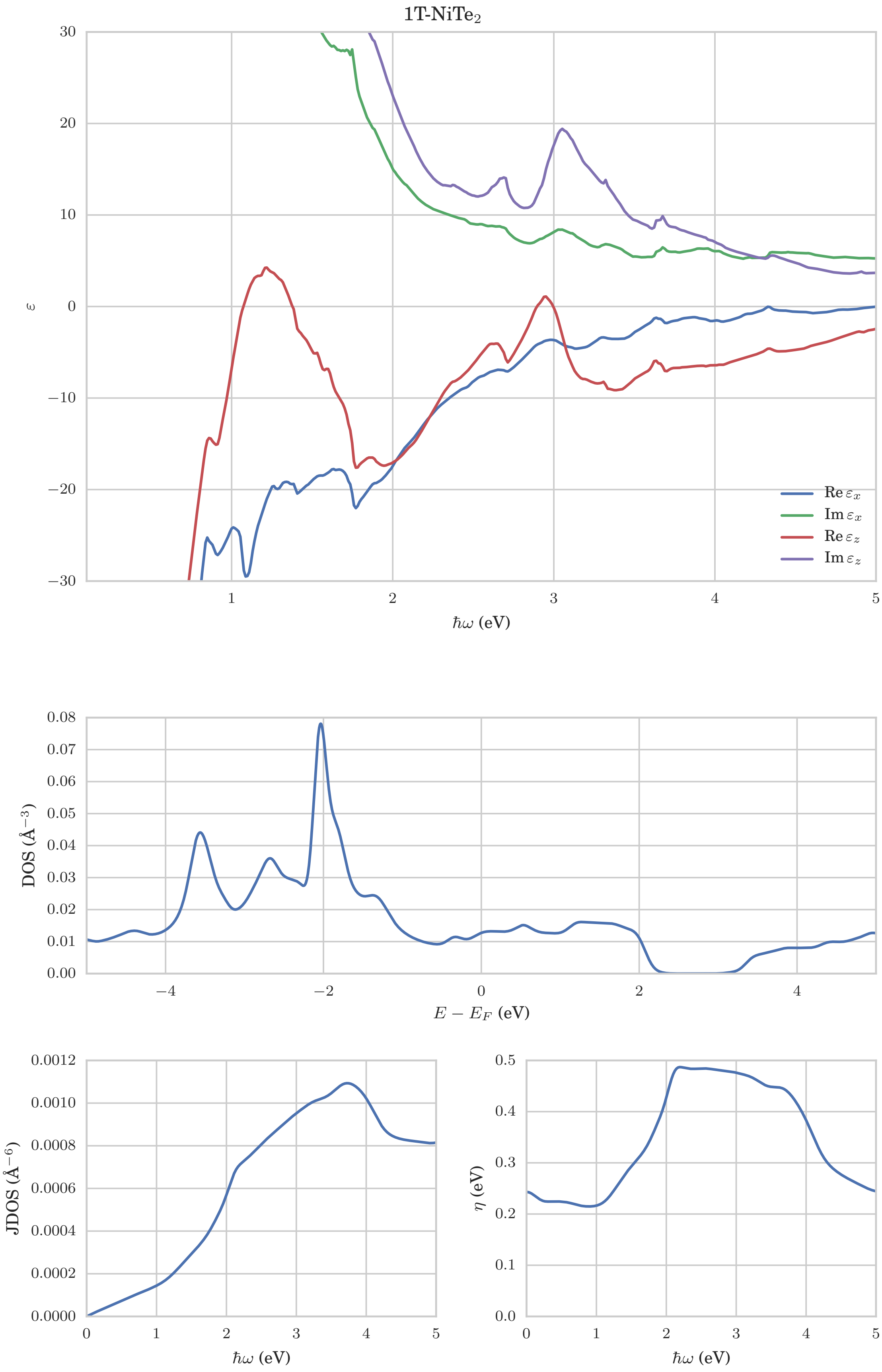

SUPPLEMENTARY FIGURE 15. **Data sheet for 1T-NiTe$_2$** Dielectric function (ε), density of states (DOS), joint density of states (JDOS) and scattering rate (η).

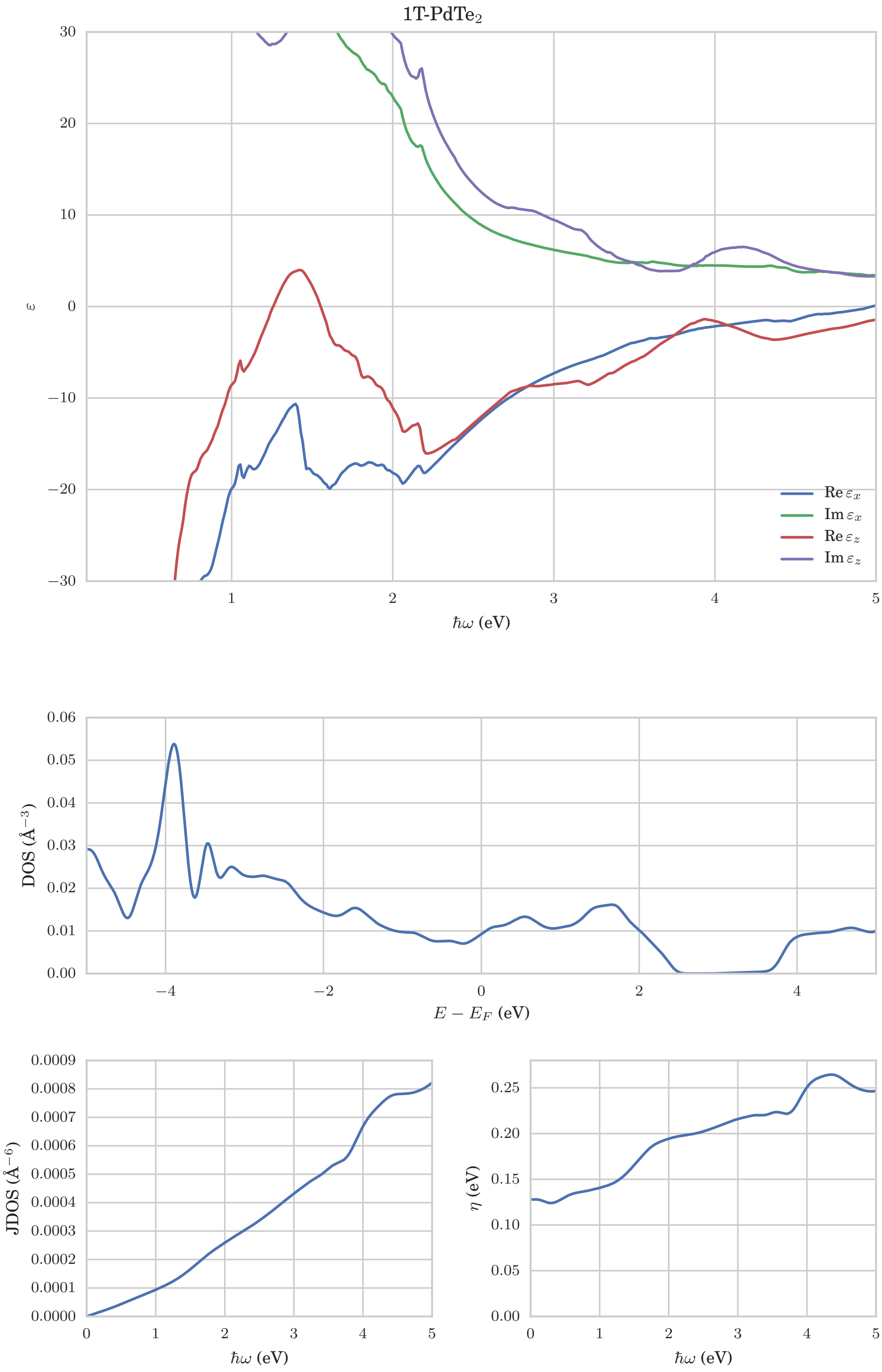

SUPPLEMENTARY FIGURE 16. **Data sheet for 1T-PdTe$_2$** Dielectric function (ε), density of states (DOS), joint density of states (JDOS) and scattering rate (η).

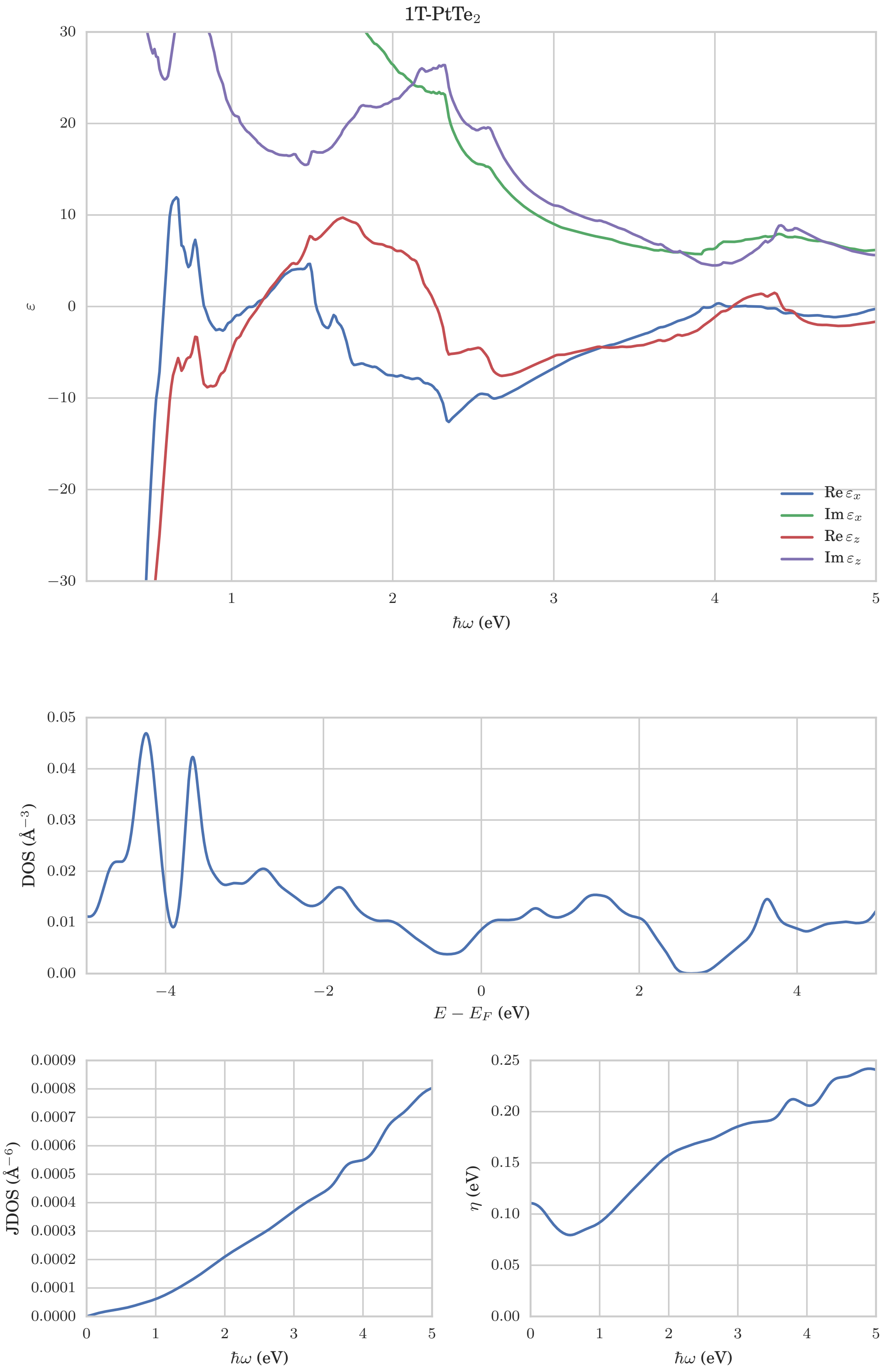

SUPPLEMENTARY FIGURE 17. **Data sheet for 1T-PtTe**$_2$ Dielectric function (ε), density of states (DOS), joint density of states (JDOS) and scattering rate (η).

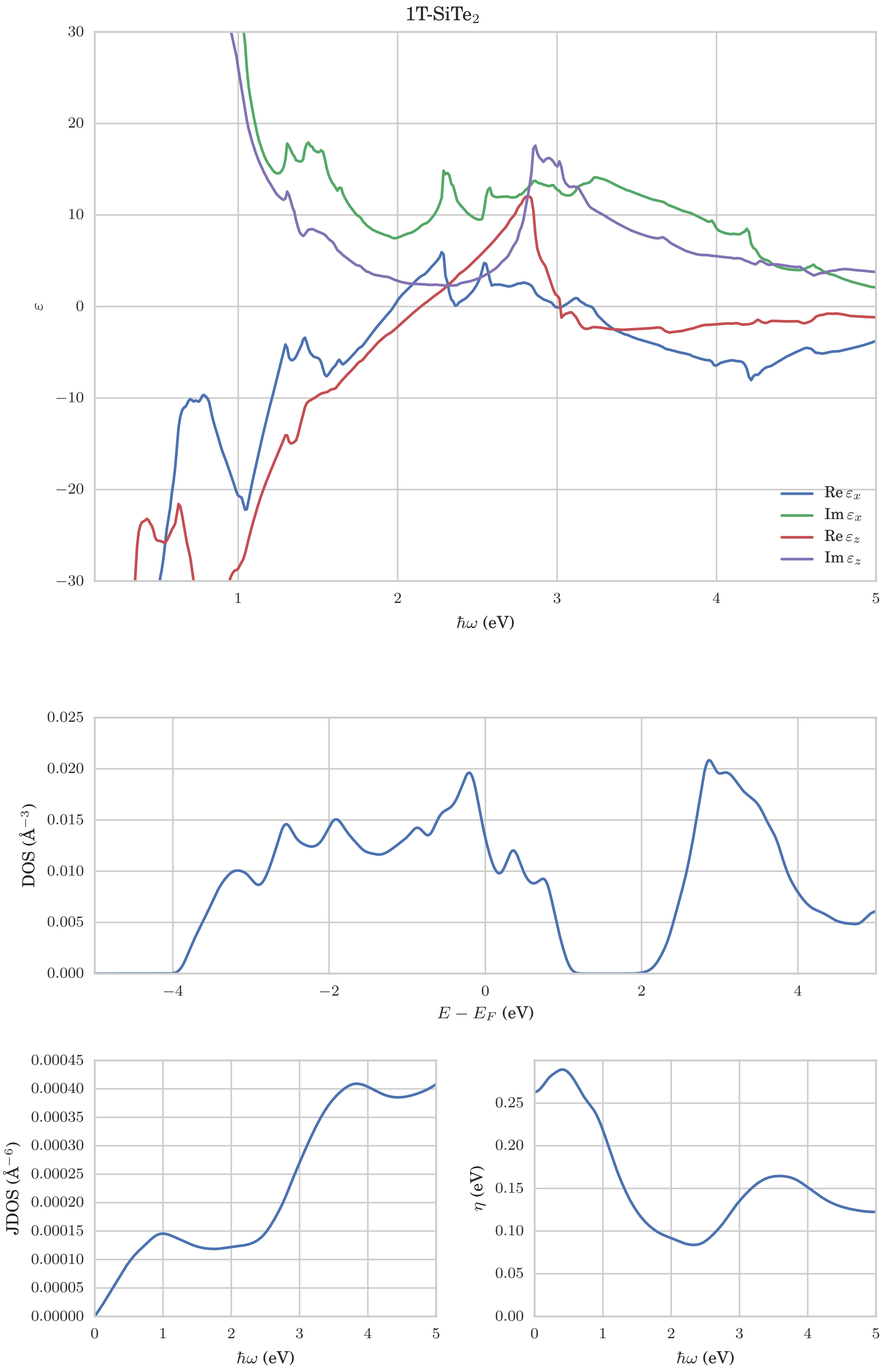

SUPPLEMENTARY FIGURE 18. **Data sheet for 1T-SiTe$_2$** Dielectric function (ε), density of states (DOS), joint density of states (JDOS) and scattering rate (η).

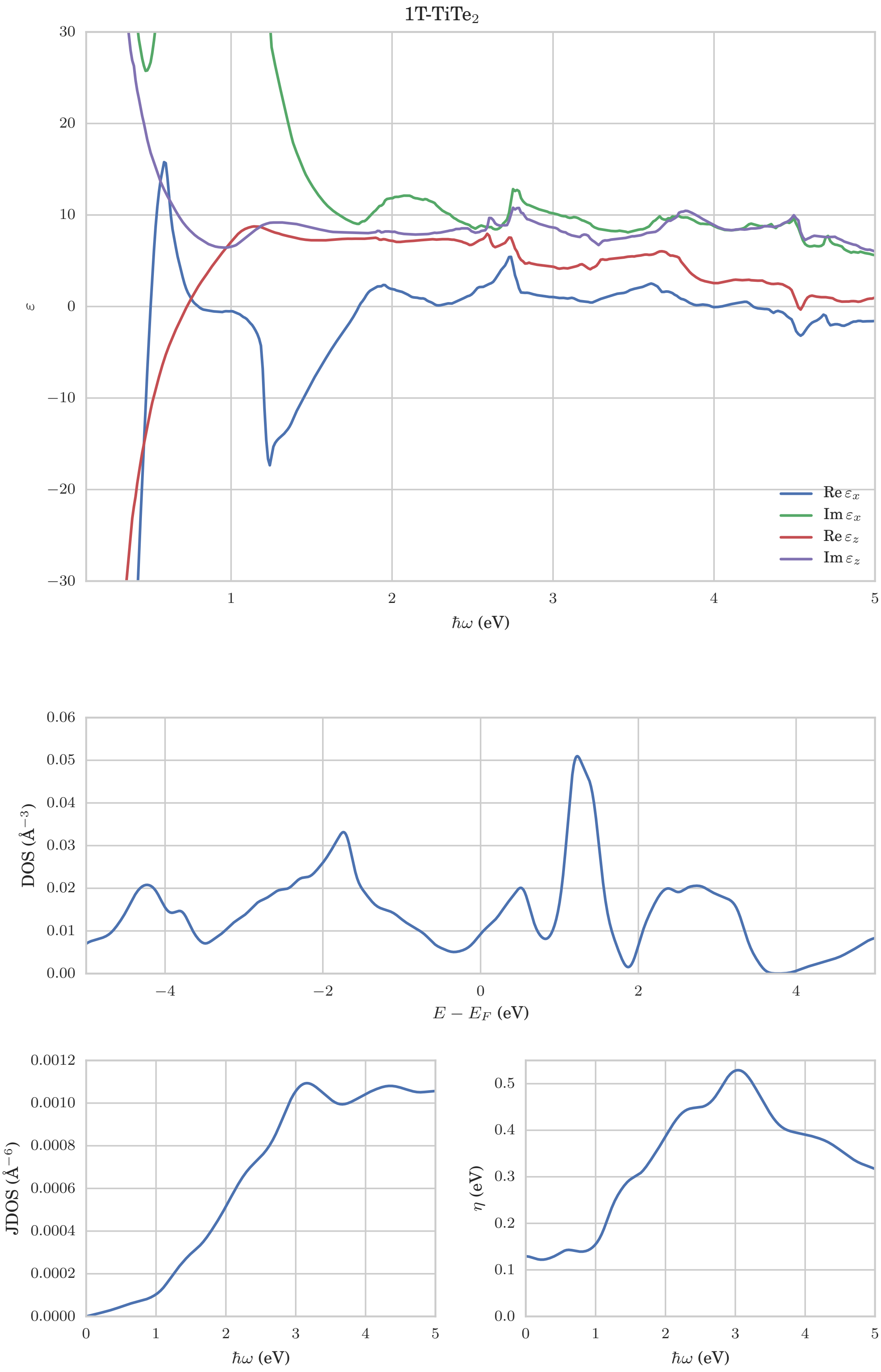

SUPPLEMENTARY FIGURE 19. **Data sheet for 1T-TiTe$_2$** Dielectric function (ε), density of states (DOS), joint density of states (JDOS) and scattering rate (η).

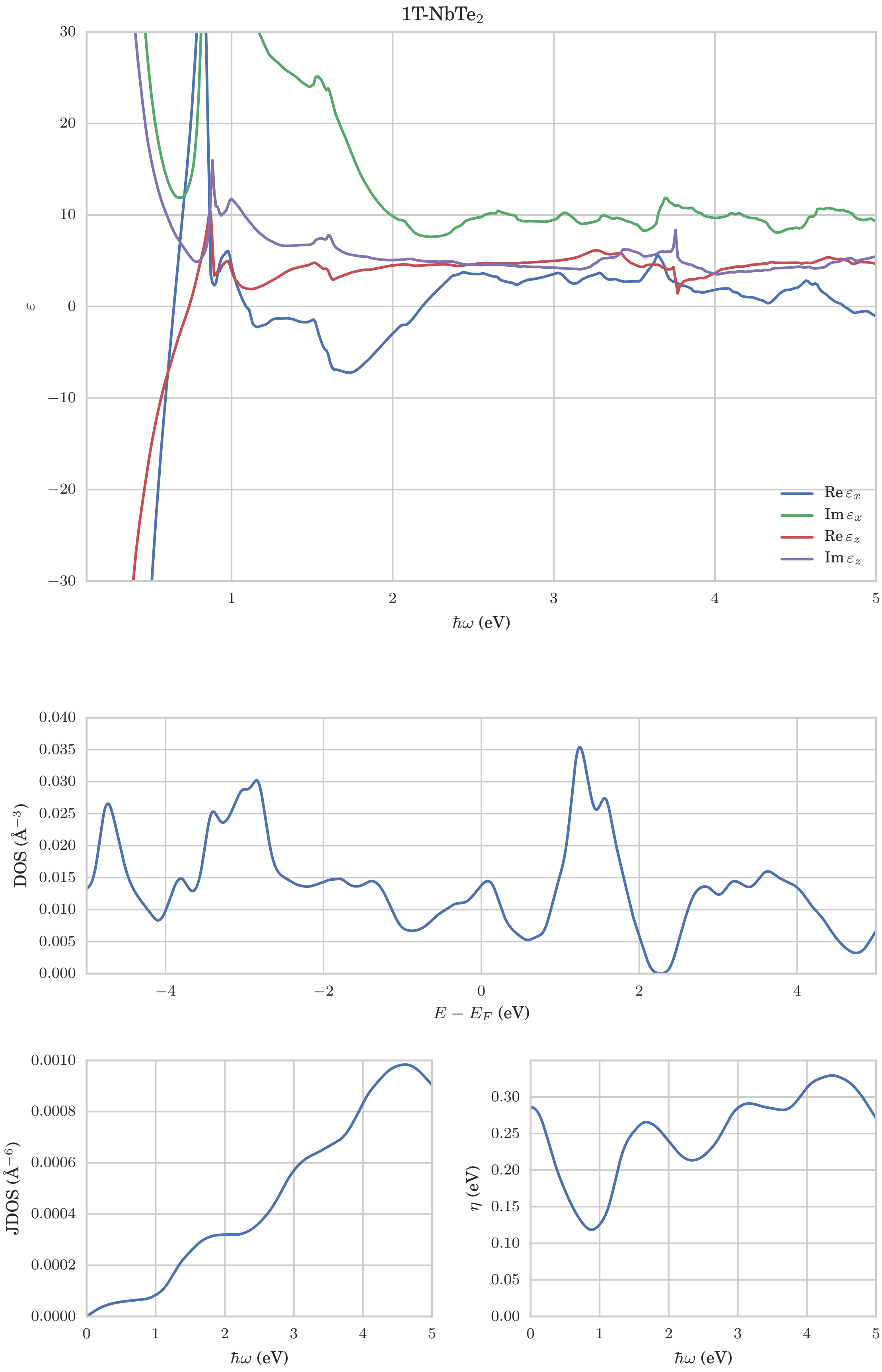

SUPPLEMENTARY FIGURE 20. **Data sheet for 1T-NbTe$_2$** Dielectric function (ε), density of states (DOS), joint density of states (JDOS) and scattering rate (η).

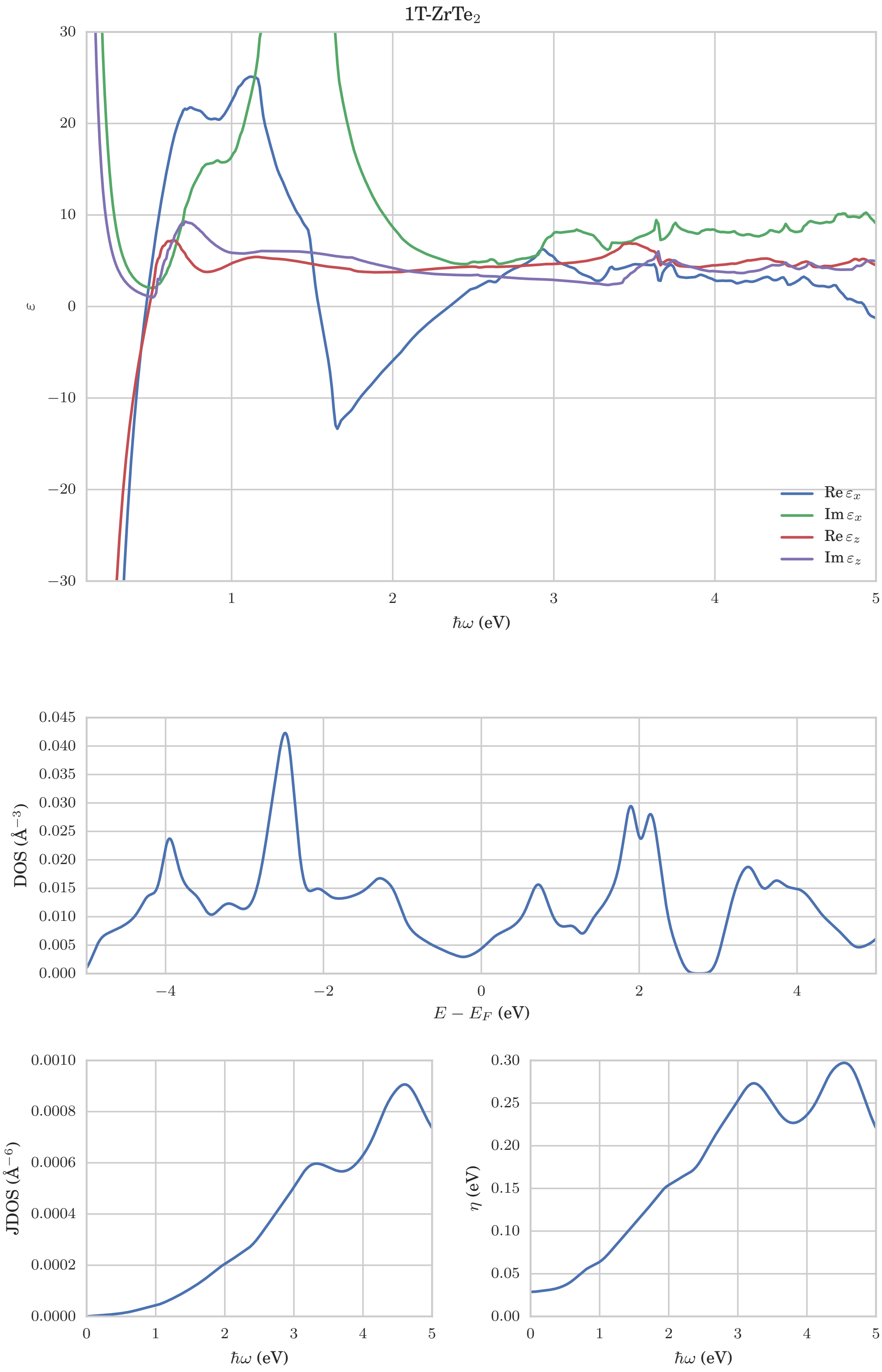

SUPPLEMENTARY FIGURE 21. **Data sheet for 1T-ZrTe$_2$** Dielectric function (ε), density of states (DOS), joint density of states (JDOS) and scattering rate (η).

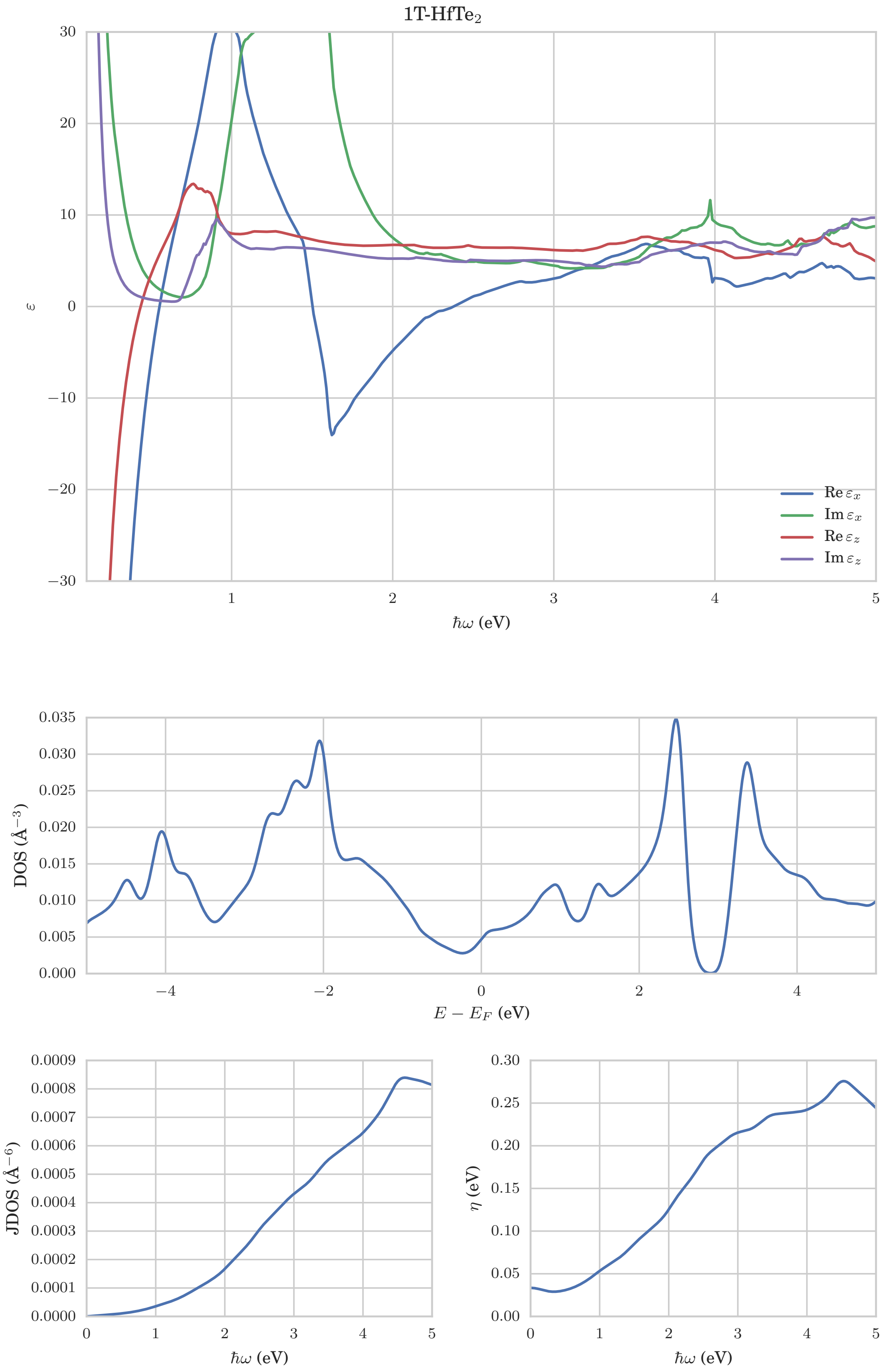

SUPPLEMENTARY FIGURE 22. **Data sheet for 1T-HfTe$_2$** Dielectric function (ε), density of states (DOS), joint density of states (JDOS) and scattering rate (η).

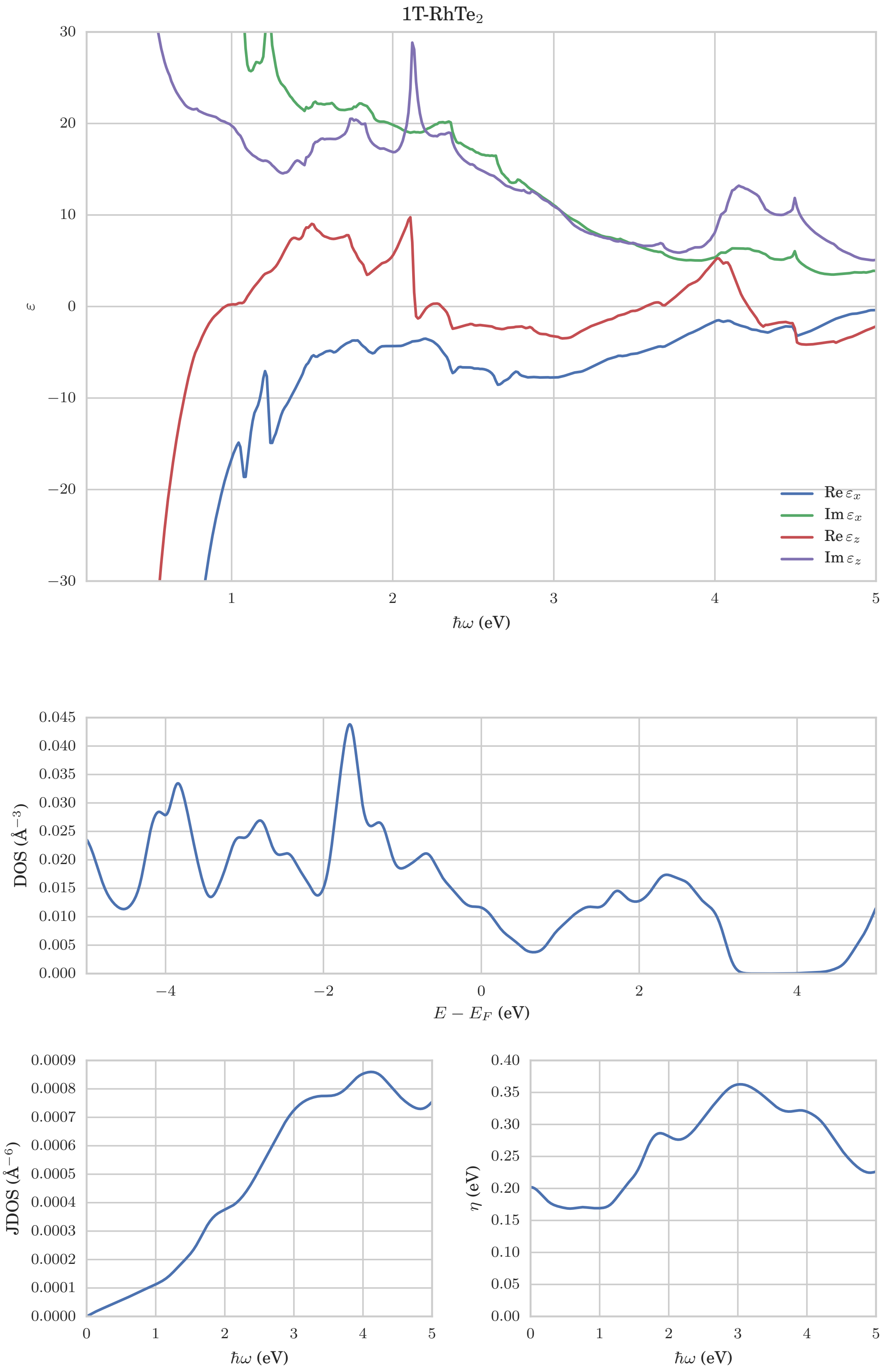

SUPPLEMENTARY FIGURE 23. **Data sheet for 1T-RhTe$_2$** Dielectric function (ε), density of states (DOS), joint density of states (JDOS) and scattering rate (η).

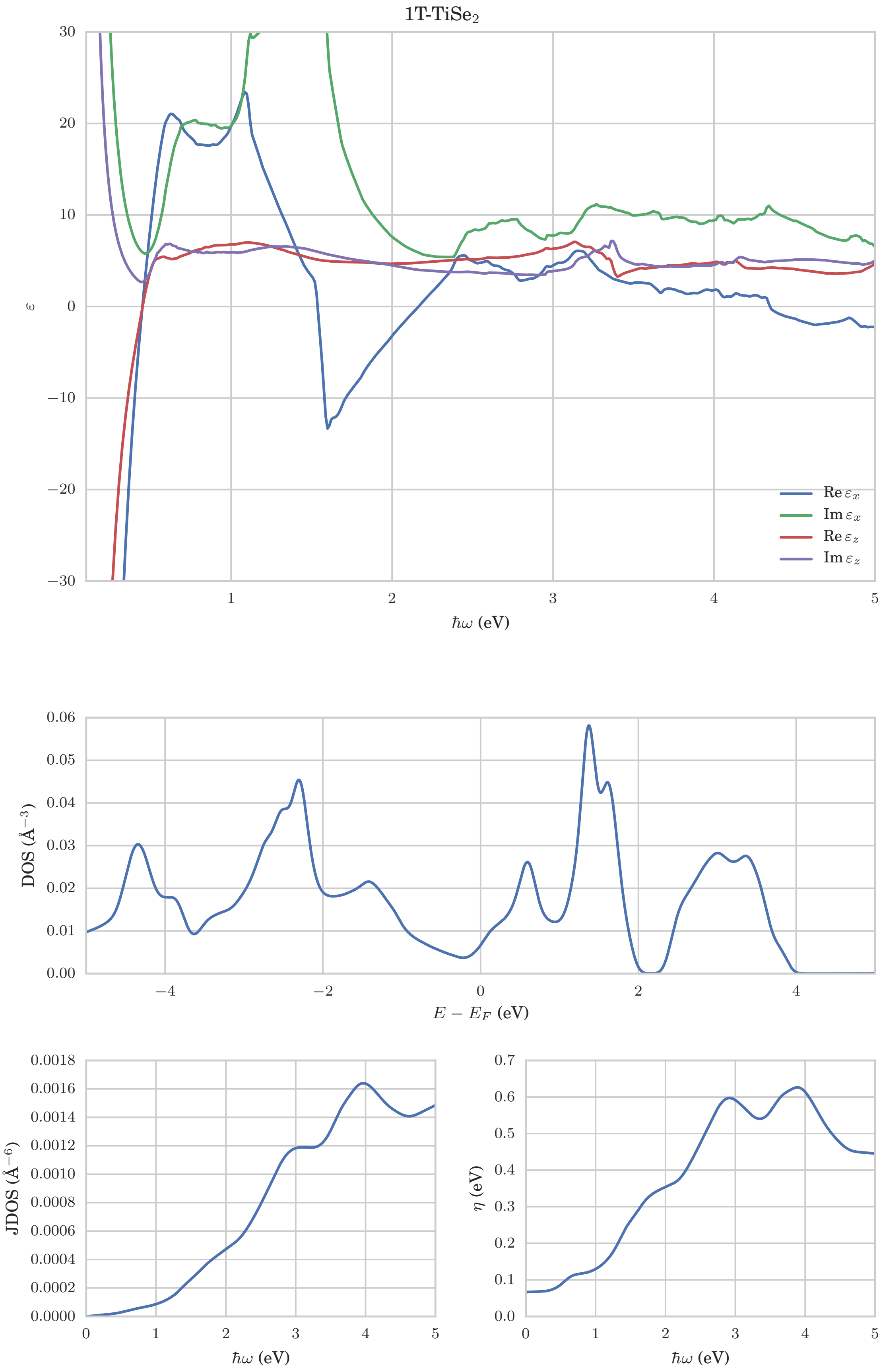

SUPPLEMENTARY FIGURE 24. **Data sheet for 1T-TiSe$_2$** Dielectric function (ε), density of states (DOS), joint density of states (JDOS) and scattering rate (η).

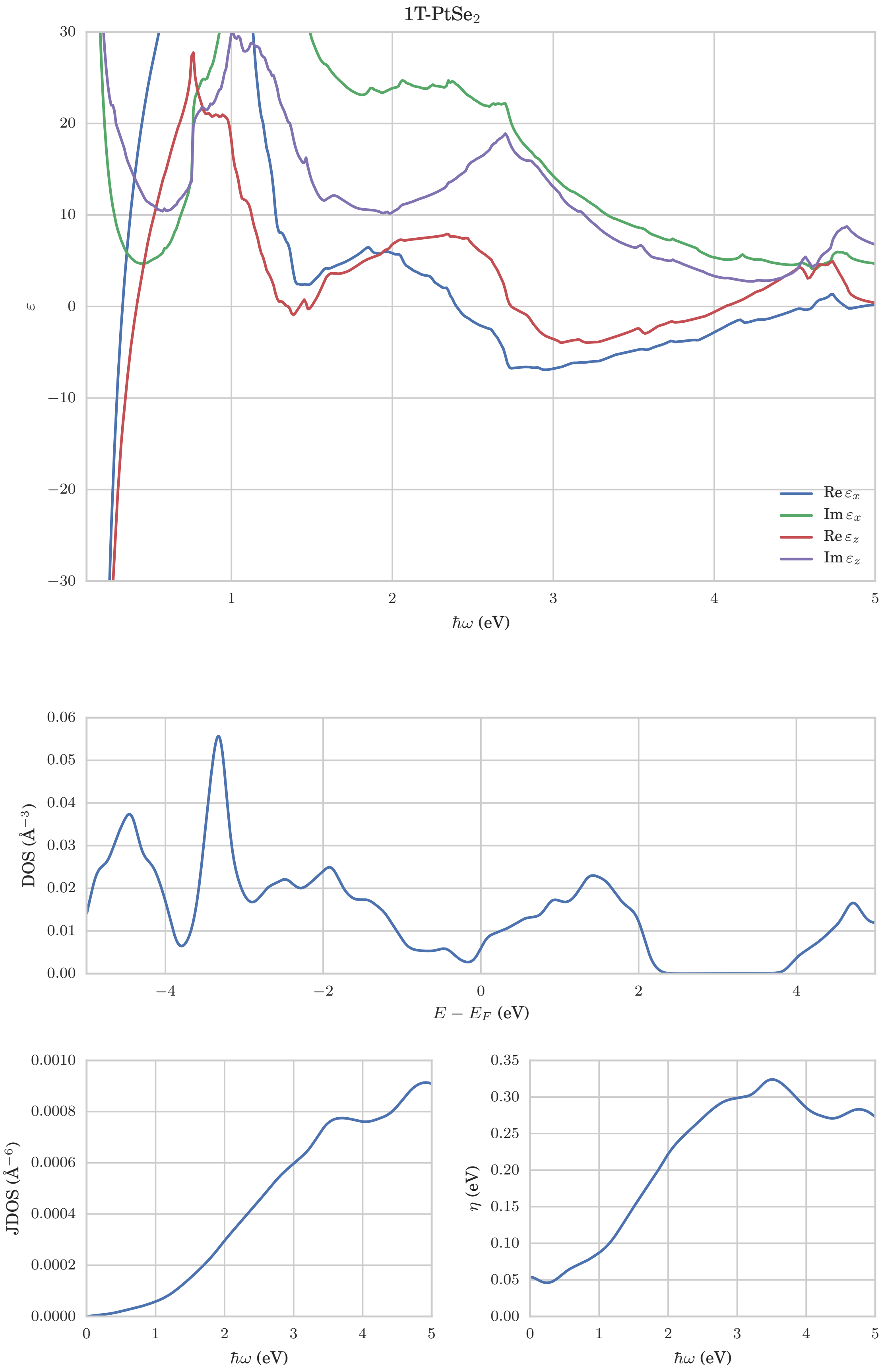

SUPPLEMENTARY FIGURE 25. **Data sheet for 1T-PtSe$_2$** Dielectric function (ε), density of states (DOS), joint density of states (JDOS) and scattering rate (η).

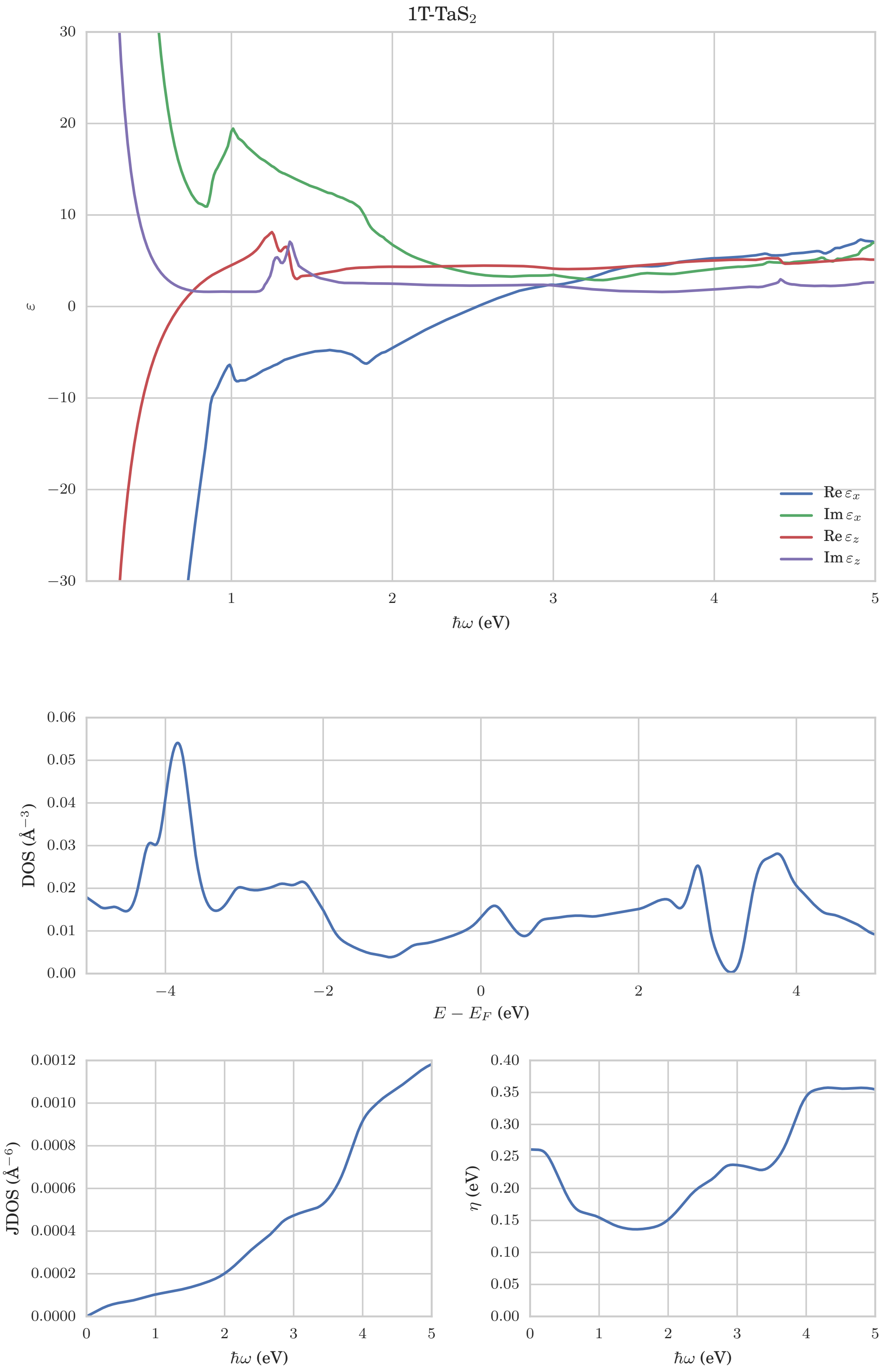

SUPPLEMENTARY FIGURE 26. **Data sheet for 1T-TaS$_2$** Dielectric function (ε), density of states (DOS), joint density of states (JDOS) and scattering rate (η).

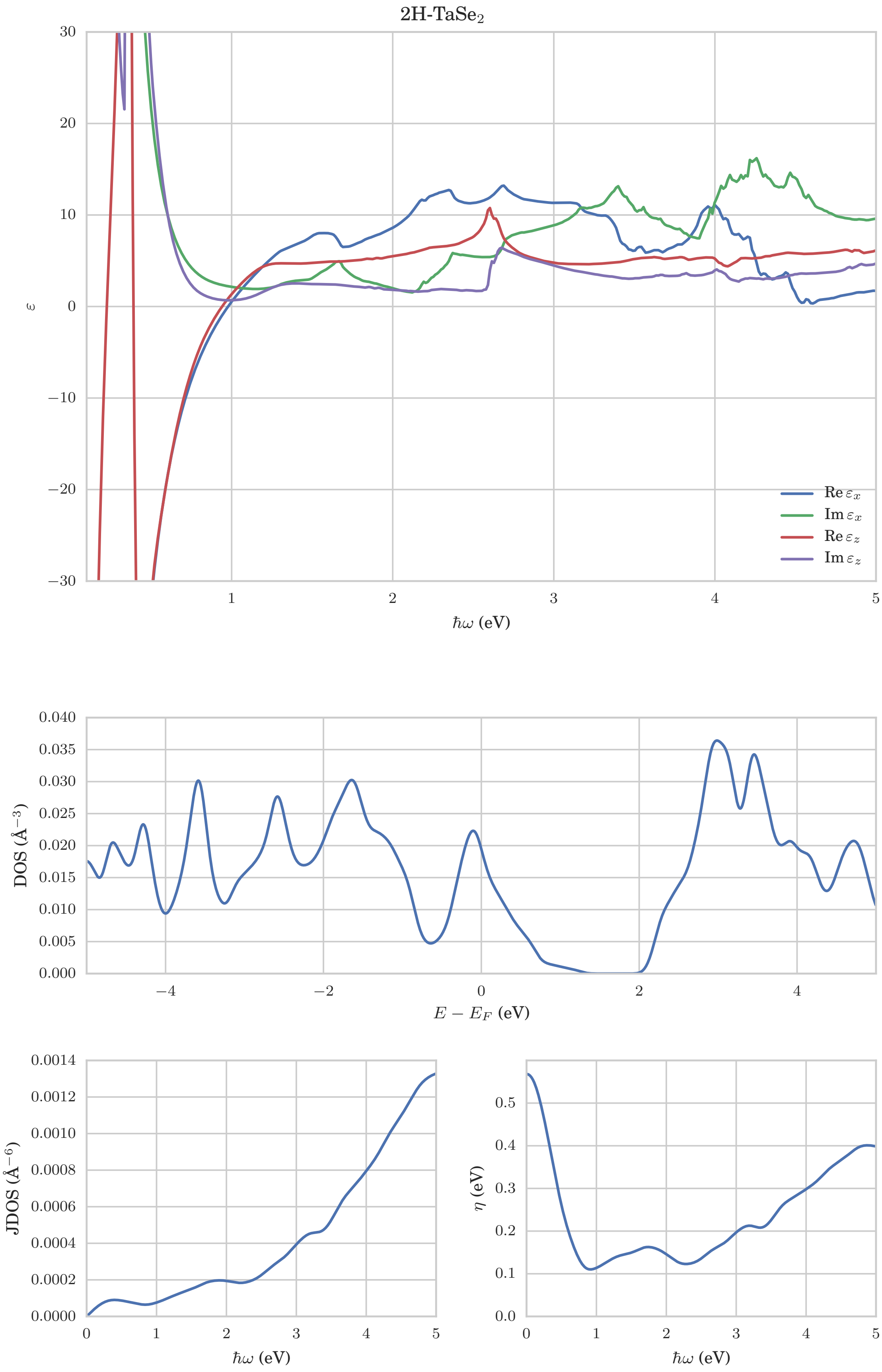

SUPPLEMENTARY FIGURE 27. **Data sheet for 2H-TaSe$_2$** Dielectric function (ε), density of states (DOS), joint density of states (JDOS) and scattering rate (η).

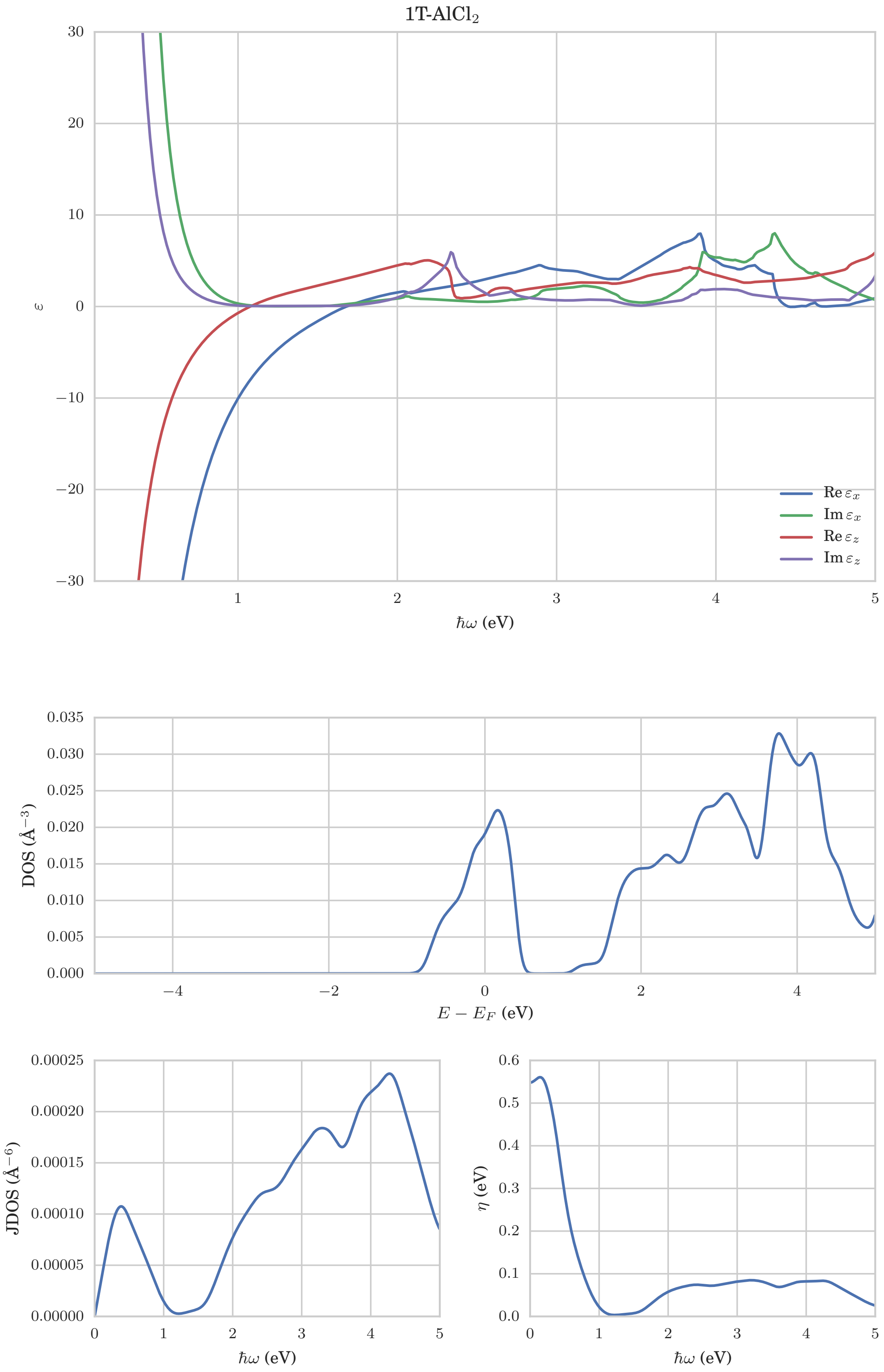

SUPPLEMENTARY FIGURE 28. **Data sheet for 1T-AlCl$_2$** Dielectric function (ε), density of states (DOS), joint density of states (JDOS) and scattering rate (η).

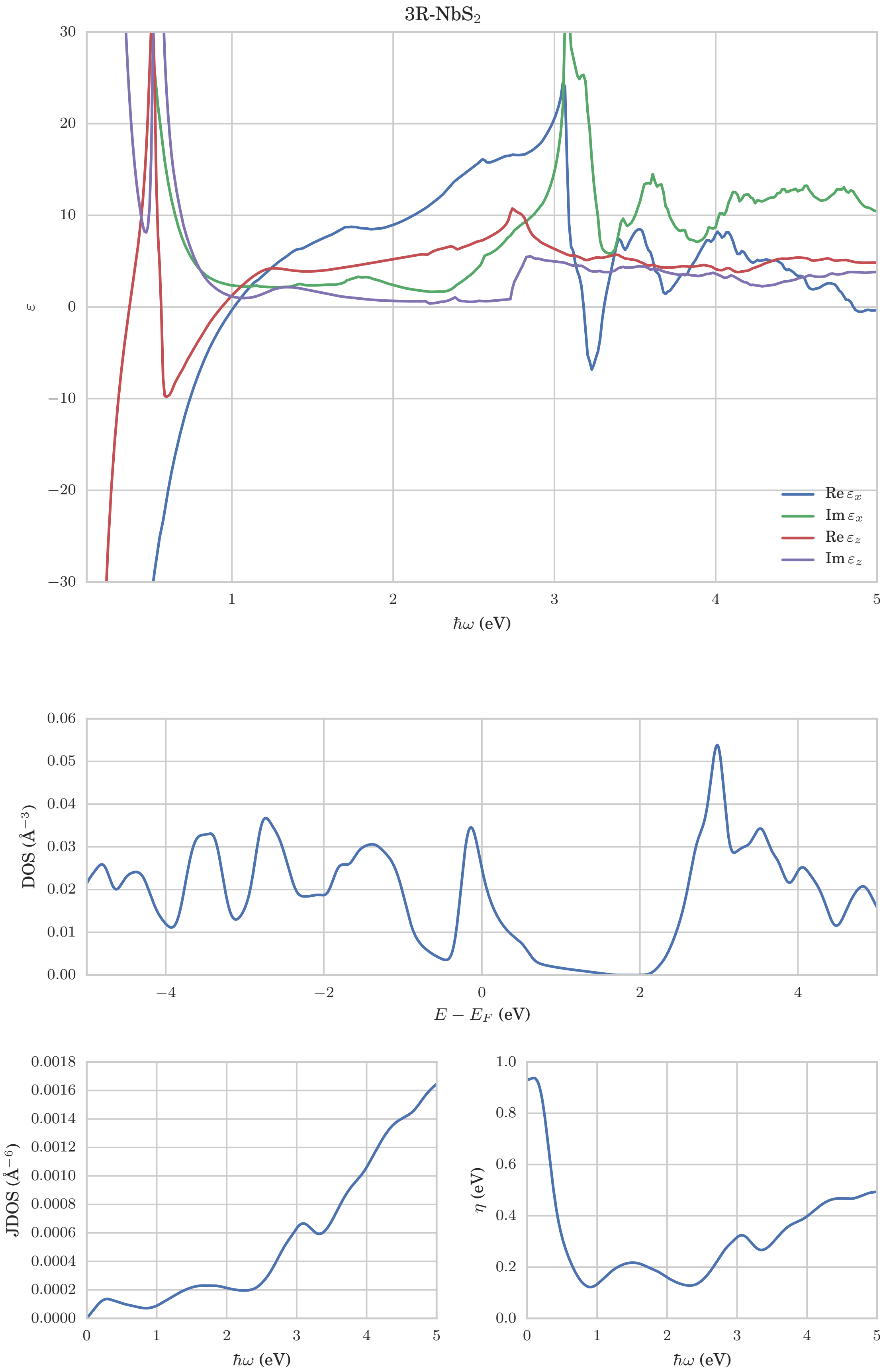

SUPPLEMENTARY FIGURE 29. **Data sheet for 3R-NbS$_2$** Dielectric function (ε), density of states (DOS), joint density of states (JDOS) and scattering rate (η).

SUPPLEMENTARY REFERENCES

[1] Lebègue, S., Björkman, T., Klintenberg, M., Nieminen, R. M. & Eriksson, O. Two-dimensional materials from data filtering and Ab Initio calculations. *Physical Review X* **3**, 1–7 (2013).

[2] Pandey, M. & Jacobsen, K. W. Heats of formation of solids with error estimation: The mBEEF functional with and without fitted reference energies. *Physical Review B* **91**, 235201 (2015).
